# Supplementary material for: Light-regulated allosteric switch enables temporal and subcellular control of enzyme activity
Source: eLife. 2020 Sep 23;9:e60647. doi: 10.7554/eLife.60647 (PMC7577742; doi:10.7554/eLife.60647)
Supplement: Supplementary file 1. [file elife-60647-supp1.docx]

atggtgcccaagaagaagaggaaagtctccaacctgctgactgtgcaccaaaacctgcctgccctccctgtggatgccac

ctctgatgaagtcaggaagaacctgatggacatgttcagggacaggcaggccttctctgaacacacctggaagatgctcc

tgtctgtgtgcagatcctgggctgcctggtgcaagctgaacaacaggaaatggttccctgctgaacctgaggatgtgagg

gactacctcctgtacctgcaagccagaggcctggctgtgaagaccatccaacagcacctgggccagctcaacatgctgca

caggagatctggcctgcctcgcccttctgactccaatgctgtgtccctggtgatgaggagaatcagaaaggagaatgtgg

atgctggggagagagccaagcaggccctggcctttgaacgcactgactttgaccaagtcagatccctgatggagaactct

ggaccaggtGGCAGCGGAGGTCATACCtTgTAtGCGCCGGGGGGTTATGACATCATGGGTTACCTCATACAGATCATGAA

taGGCCGAACCCACAAGTGGAGCTcGGACCCGTCGAtACCTCCTGCGCtCTCATtCTGTGTGACCTTAAGCAGAAGGAtA

CCCCTaTcGTGTACGCCTCCGAgGCATTtCTGTACATGACaGGGTACTCGAACGCCGAAGTGCTGGGACGGAACTGCCGC

TTCCTGCAAagCCCGGATGGAATGGTGAAGCCTAAGTCAACCCGCAAATACGTGGACTCCAACACTATCAACACCATGCG

CAAGGCCATTGACCGCAATGCTGAGGTGCAAGTGGAAGTGGTGAACTTCAAGAAGAAtGGACAGCGCTTCGTCAACTTCC

TGACTATGATcCCCGTGCGcGACGAgACCGGCGAATACCGGTACAGCATGGGGTTTCAGTGtGAGACaGAGGGCGGCTCC

GGAGGCAGCGGCGGTTCTGGAGGTTCCGGTGGCGGCTCCGGAGGTAGCGGAGGGTCTCACACTCTTTACGCCCCTGGAGG

ATACGACATTATGGGATATTTGATTCAGATTATGAACCGCCCAAACCCTCAGGTCGAACTGGGGCCTGTGGACACGTCAT

GTGCCCTGATCCTGTGCGATCTGAAGCAAAAGGACACTCCGATTGTCTACGCCTCGGAAGCCTTCTTGTATATGACCGGA

TACAGCAATGCAGAGGTGCTCGGTAGGAACTGCAGATTCCTGCAGTCCCCCGACGGGATGGTGAAACCAAAGTCGACTCG

CAAATATGTGGACTCGAACACGATCAAtACaATgCGGAAGGCCATCGACCGGAACGCCGAGGTCCAGGTGGAGGTGGTCA

ACTTTAAGAAGAACGGCCAGCGGTTCGTGAACTTTCTcACCATgATTCCGGTCCGGGATGAAACCGGAGAGTACAGATAC

TCCATGGGATTCCAGTGCGAAACCGAAGGGTCCGGAggtcccggaggt

agatgccaggacatcaggaacctggccttcctgggcattgcctacaacaccctgctgcgcattgccgaaattgccagaat

cagagtgaaggacatctcccgcaccgatggtgggagaatgctgatccacattggcaggaccaagaccctggtgtccacag

ctggtgtggagaaggccctgtccctgggggttaccaagctggtggagagatggatctctgtgtctggtgtggctgatgac

cccaacaactacctgttctgccgggtcagaaagaatggtgtggctgccccttctgccacctcccaactgtccacccgggc

cctggaagggatctttgaggccacccaccgcctgatctatggtgccaaggatgactctgggcagagatacctggcctggt

ctggccactctgccagagtgggtgctgccagggacatggccagggctggtgtgtccatccctgaaatcatgcaggctggt

ggctggaccaatgtgaacattgtgatgaactacatcagaaacctggactctgagactggggccatggtgaggctgctcga

ggatggggacAgaattctgcagtcgacggtaccgcgggcccgggatccaccggtcgccacc

atggtagcaggtcatgcctctggcagccccgcattcgggaccgcctctcattcgaattgcgaacatgaagagatccacct

cgccggctcgatccagccgcatggcgcgcttctggtcgtcagcgaacatgatcatcgcgtcatccaggccagcgccaacg

ccgcggaatttctgaatctcggaagcgtactcggcgttccgctcgccgagatcgacggcgatctgttgatcaagatcctg

ccgcatctcgatcccaccgccgaaggcatgccggtcgcggtgcgctgccggatcggcaatccctctacggagtactgcgg

tctgatgcatcggcctccggaaggcgggctgatcatcgaactcgaacgtgccggcccgtcgatcgatctgtcaggcacgc

tggcgccggcgctggagcggatccgcacggcgggttcactgcgcgcgctgtgcgatgacaccgtgctgctgtttcagcag

tgcaccggctacgaccgggtgatggtgtatcgtttcgatgagcaaggccacggcctggtattctccgagtgccatgtgcc

tgggctcgaatcctatttcggcaaccgctatccgtcgtcgactgtcccgcagatggcgcggcagctgtacgtgcggcagc

gcgtccgcgtgctggtcgacgtcacctatcagccggtgccgctggagccgcggctgtcgccgctgaccgggcgcgatctc

gacatgtcgggctgcttcctgcgctcgatgtcgccgtgccatctgcagttcctgaaggacatgggcgtgcgcgccaccct

ggcggtgtcgctggtggtcggcggcaagctgtggggcctggttgtctgtcaccattatctgccgcgcttcatccgtttcg

agctgcgggcgatctgcaaacggctcgccgaaaggatcgcgacgcggatcaccgcgcttgagagc
