# Supplementary material for: Light-regulated allosteric switch enables temporal and subcellular control of enzyme activity
Source: eLife. 2020 Sep 23;9:e60647. doi: 10.7554/eLife.60647 (PMC7577742; doi:10.7554/eLife.60647)
Supplement: Transparent reporting form [file elife-60647-transrepform.docx]

***eLife’s* transparent reporting form**

We encourage authors to provide detailed information *within their submission* to facilitate the interpretation and replication of experiments. Authors can upload supporting documentation to indicate the use of appropriate reporting guidelines for health-related research (see [EQUATOR Network](http://www.equator-network.org/%20)), life science research (see the [BioSharing Information Resource](https://biosharing.org/)), or the [ARRIVE guidelines](http://www.plosbiology.org/article/info:doi/10.1371/journal.pbio.1000412) for reporting work involving animal research. Where applicable, authors should refer to any relevant reporting standards documents in this form.

If you have any questions, please consult our Journal Policies and/or contact us: [editorial@elifesciences.org](mailto:editorial@elifesciences.org).

**Sample-size estimation**

- You should state whether an appropriate sample size was computed when the study was being designed
- You should state the statistical method of sample size computation and any required assumptions
- If no explicit power analysis was used, you should describe how you decided what sample (replicate) size (number) to use

Please outline where this information can be found within the submission (e.g., sections or figure legends), or explain why this information doesn’t apply to your submission:

Sample size was determined based on previous studies that used similar analysis (PMID: 24609359 and PMID:19693014).

Sample size for the experiments presented is indicated in the figure legends.

**Replicates**

- You should report how often each experiment was performed
- You should include a definition of biological versus technical replication
- The data obtained should be provided and sufficient information should be provided to indicate the number of independent biological and/or technical replicates
- If you encountered any outliers, you should describe how these were handled
- Criteria for exclusion/inclusion of data should be clearly stated
- High-throughput sequence data should be uploaded before submission, with a private link for reviewers provided (these are available from both GEO and ArrayExpress)

Please outline where this information can be found within the submission (e.g., sections or figure legends), or explain why this information doesn’t apply to your submission:

Molecular dynamic simulations were performed 3 times, each at 100 ns.

All remaining experiments were performed at least three times, on different days, with similar results.

Biological replicates are separate and independent experiments performed at different times in the same exact way.

Technical replicates are multiple analysis of the same biological sample.

No data was excluded from the analysis.

Number of replicates for the experiments presented is indicated in the figure legend.

**Statistical reporting**

- Statistical analysis methods should be described and justified
- Raw data should be presented in figures whenever informative to do so (typically when N per group is less than 10)
- For each experiment, you should identify the statistical tests used, exact values of N, definitions of center, methods of multiple test correction, and dispersion and precision measures (e.g., mean, median, SD, SEM, confidence intervals; and, for the major substantive results, a measure of effect size (e.g., Pearson's r, Cohen's d)
- Report exact p-values wherever possible alongside the summary statistics and 95% confidence intervals. These should be reported for all key questions and not only when the p-value is less than 0.05.

Please outline where this information can be found within the submission (e.g., sections or figure legends), or explain why this information doesn’t apply to your submission:

Statistical analysis methods for the different studies performed are described in detail under the Materials and Methods section.

All experiments presented include their number of repeats and the exact values of N in their relative figure legends.

The dispersion and precision measures of all graphs presented are identified in their relative figure legends.

(For large datasets, or papers with a very large number of statistical tests, you may upload a single table file with tests, Ns, etc., with reference to sections in the manuscript.)

**Group allocation**

- Indicate how samples were allocated into experimental groups (in the case of clinical studies, please specify allocation to treatment method); if randomization was used, please also state if restricted randomization was applied
- Indicate if masking was used during group allocation, data collection and/or data analysis

Please outline where this information can be found within the submission (e.g., sections or figure legends), or explain why this information doesn’t apply to your submission:

This information does not apply to our studies, since no comparison between different groups was performed.

**Additional data files (“source data”)**

- We encourage you to upload relevant additional data files, such as numerical data that are represented as a graph in a figure, or as a summary table
- Where provided, these should be in the most useful format, and they can be uploaded as “Source data” files linked to a main figure or table
- Include model definition files including the full list of parameters used
- Include code used for data analysis (e.g., R, MatLab)
- Avoid stating that data files are “available upon request”

Please indicate the figures or tables for which source data files have been provided:

Metamorph software version 7.8.2.0 was used to collect fluorescent images in this study.

To analyze the mass spectrometry phosphoproteomics data, we used MATLAB software (version R2019b, Bioinformatics Toolbox version 4.13, MathWorks). Data were plotted with the ‘clustergram’ function with hierarchical clustering using Euclidean distance. PCA was performed using Scikit-learn (v0.19.1) in Python (v3.6.0). For STRING network analysis was performed using the open source platform [http://string-db.org/.](http://string-db.org/) An open source MATLAB application CellGeo (PMID: 24493591) and EdgeProps (PMID: 30062404) were used to analyze cell area and cell edge velocity. For image analysis, we also used Metamorph software version 7.8.2.0.

Dataset 1 table includes the raw data for the mass spectrometry studies performed. This data is presented in figure 2c-e, supplementary figure 2d-g, as well as supplementary figure 2b-c.
